# A Retrospective Study Examining Self-Reported Alcohol Intake Among Adult Patients With Myocardial Infarction

**DOI:** 10.7759/cureus.92276

**Published:** 2025-09-14

**Authors:** Amruth A Alluri, Pranesh Ramesh, Akanksha Gangula, Mohammad Nasim Nikzad, Shrey Chetankumar Mehta, Prathvi Alva, Pavan Bolla

**Affiliations:** 1 Internal Medicine, American University of the Caribbean School of Medicine, Cupecoy, SXM; 2 Cardiology, PSG Institute of Medical Science and Research, Coimbatore, IND; 3 Cardiology, International Higher School of Medicine, Bishkek, KGZ; 4 Cardiology, Jamhoriate Hospital, Kabul, AFG; 5 Internal Medicine, M.P. Shah Medical College, Jamnagar, IND; 6 Cardiology, Srinivas Institute of Medical Sciences & Research Centre, Mangalore, IND; 7 Primary Care, Westchester Independent and Assisted Living, Yonkers, USA

**Keywords:** binge drinking, brfss, myocardial infarction, odd ratio, retrospective study

## Abstract

Introduction: Alcohol intake has long been linked to cardiovascular risk, but prior evidence is inconsistent. While chronic heavy use is associated with increased risk of myocardial infarction (MI) and coronary heart disease (CHD), some studies suggest protective effects of moderate consumption. The impact of binge drinking, a distinct and common drinking pattern, on MI and CHD remains less clearly defined. Understanding this association is important for refining prevention and public health strategies.

Aim: This study aims to evaluate the association between self-reported binge drinking (as defined by the CDC (Centers for Disease Control and Prevention) criteria) and the prevalence of MI or CHD in adults, and to assess whether this association varies across demographic and socioeconomic subgroups using the 2022 Behavioral Risk Factor Surveillance System (BRFSS) dataset.

Methodology: This retrospective study utilized the BRFSS database. The disease variable was self-reported MI, and the risk factor was binge drinking (yes/no). Control variables included age, gender, race, and socioeconomic factors (education and income). Data were analyzed using cross-tabulations and tests, with results expressed as odds ratios (ORs) and 95% confidence intervals (CIs).

Results: Out of 56,539 binge drinkers, 2,570 (4.5%) had MI, and out of 333,406 non-binge drinkers, 300,336 (90.1%) did not have MI. The odds ratio of 0.432 indicates that participants who reported being binge drinkers were 56.8% less likely to have MI when compared to non-binge drinkers. MI was more prevalent in males, with 1,942 of 34,272 binge drinkers (5.7%), compared with 628 of 22,267 binge drinkers (2.8%) in females. About 4.6% of 41,547 White binge drinkers had MI, 4.9% of 3,253 Black binge drinkers had MI, and 3.5% of 6,331 Hispanic binge drinkers had MI. The likelihood of association between MI or CHD and binge drinking was 46.9% higher (OR=1.469) in those aged 18-24 years, 24.3% lower (OR=0.757) in those aged 25-44 years, 27.6% lower (OR=0.724) in those aged 45-64 years, and 18.8% lower (OR=0.812) in those aged 65 years and older. The likelihood of association between MI or CHD and binge drinking was 58.5% lower (OR=0.415) in males, and 65.3% lower (OR=0.347) in females. The likelihood of association between MI or CHD and binge drinking was 59.5% lower (OR=0.405) in White participants, 42.9% lower (OR=0.571) in Black participants, and 45.5% lower (OR=0.545) in Hispanic participants. Prevalence of MI or CHD in binge drinkers was 983 (6.1%) among participants with basic education out of a total of 16,023 and 1,578 (3.9%) among participants with advanced education out of a total of 40,373. The likelihood of association of MI or CHD in binge drinkers was 52.5% lower (OR=0.475) in participants with basic education, and 58.9% lower (OR=0.411) in participants with advanced education. The likelihood of association of MI or CHD in binge drinkers was 48.5% lower (OR=0.515) in participants with income <$50,000, and 56.9% lower (OR=0.431) in participants with income >$50,000.

Conclusion: In this cross-sectional analysis of the 2022 BRFSS dataset, binge drinking was associated with lower reported prevalence of MI/CHD. These findings should be interpreted cautiously, given the self-reported data, potential confounding, and inability to infer causality.

## Introduction

Alcohol consumption has been causally related to the incidence of coronary heart disease (CHD), but the role of alcohol before the event has not been explored in depth [1]. The mechanism behind this is not clear but may be direct (alcohol myotoxicity) or indirect (through alcohol-derived metabolites or effects on other organs such as the adrenal glands) [2]. A 2010 case-control study conducted in Kragujevac, Serbia, investigated the link between binge drinking and myocardial infarction (MI). The study included 374 participants, evenly divided between newly diagnosed MI patients and controls, matched by gender, age, and residence. Results showed that MI within one year was significantly more prevalent among binge drinkers (25.1%) than among controls (12.8%). Further analysis revealed that older adults, men, and those residing in rural areas were at increased risk of acute MI associated with binge drinking [3]. In addition, chronic alcohol abuse has several cardiovascular consequences, including increased blood pressure and arterial hypertension, altered blood coagulation, reduced cerebral blood flow and vasoconstriction, and increased risk of stroke and intracranial hemorrhage. These effects can be fatal and highlight the importance of addressing alcohol abuse to mitigate cardiovascular risk [3]. A global study spanning 52 countries found that consuming six or more drinks in a single occasion within the past 24 hours increased the risk of MI by 40%. This risk was particularly pronounced among older adults, with those over 65 years of age facing a fivefold increased risk of MI [4]. In examining the relationship of alcohol and cardiovascular health, most, but not all, epidemiological studies suggest that light to moderate alcohol consumption can reduce the incidence of coronary artery disease, ischemic stroke, and peripheral arterial disease [5].

While previous studies have reported conflicting results, with some linking alcohol consumption to increased cardiovascular risk and others suggesting potential protective effects, most have focused on overall or moderate alcohol use rather than binge drinking. The cardiovascular impact of binge drinking, a common and distinct drinking pattern, remains less well characterized. This study addresses that gap by examining the association between binge drinking and the prevalence of MI or CHD using the 2022 Behavioral Risk Factor Surveillance System (BRFSS) dataset.

Aim and objectives

To evaluate the association between binge drinking and MI or CHD by assessing the odds of developing MI or CHD in binge drinkers versus non-binge drinkers, based on demographic (age, gender, race) and socioeconomic characteristics (annual income and education levels) of participants using the BRFSS database.

## Materials and methods

This study was a retrospective, cross-sectional analysis utilizing data from the 2022 BRFSS, a nationwide telephone survey conducted annually by the Centers for Disease Control and Prevention (CDC) [6]. The BRFSS collects self-reported information on health behaviors, chronic conditions, and preventive health measures from US adults aged 18 years and older. The dataset is publicly available and de-identified; therefore, this study was classified as non-human subject research and was exempt from Institutional Review Board (IRB) approval [7].

Exposure variable

The primary exposure was binge drinking, defined according to CDC/BRFSS criteria as consuming ≥5 drinks per occasion for men and ≥4 drinks per occasion for women, at least once in the past 30 days [6]. Broader alcohol consumption patterns (light/moderate drinking, frequency of use) were not analyzed, as they are not uniformly captured in the BRFSS dataset.

Outcome variable

The outcome was a combined endpoint of MI or CHD, determined by affirmative responses to the BRFSS questions on these conditions. MI and CHD were not analyzed separately.

Both exposure and outcome measures were self-reported and not validated against clinical records, introducing the potential for recall or misclassification bias. Important covariates such as smoking status, hypertension, diabetes, and physical activity were not available in WEAT, limiting adjustment for confounding. The cross-sectional design prevents inference of temporal or causal relationships.

Covariates

Demographic and socioeconomic factors were included to assess subgroup differences: age (18-24, 25-44, 45-64, ≥65 years); gender (male, female); race/ethnicity (White non-Hispanic, Black non-Hispanic, Hispanic/Other); education (basic education, defined as high school or less, and advanced education, defined as college or higher); and annual income (<$50,000 and ≥$50,000), consistent with BRFSS groupings. These categories were selected because they represent standard BRFSS socioeconomic indicators and allow sufficient sample sizes for analysis.

Data extraction and weighting

Data were extracted using the Web-Enabled Analysis Tool (WEAT) on the CDC BRFSS portal. WEAT incorporates BRFSS survey weights, ensuring national representativeness of the estimates. Although WEAT limits flexibility compared with direct dataset download, it provides standardized, transparent access for secondary analysis.

Statistical analysis

Descriptive statistics were used to summarize participant characteristics, prevalence of binge drinking, and prevalence of MI/CHD. Associations between binge drinking and MI/CHD were examined using stratified odds ratios (ORs) with 95% confidence intervals (CIs). All reported ORs are crude, unadjusted estimates derived from subgroup comparisons; multivariable logistic regression was not applied due to limitations of the WEAT platform. Subgroup analyses across age, gender, race/ethnicity, education, and income were exploratory and not pre-specified. No formal correction for multiple comparisons was performed; this increases the potential for type I error, which is acknowledged in the Limitations section.

Handling of missing data

Participants with missing responses for exposure, outcome, or subgroup variables were excluded on a case-wise basis. Missingness was not imputed.

## Results

The total number of people who participated in the BRFSS study in 2022 from all locations was 389,945, and no participants were excluded. The prevalence of MI or CHD differed between binge drinkers and non-binge drinkers. Out of 56,539 binge drinkers, 2,570 (4.5%) had MI or CHD, and out of 333,406 non-binge drinkers, 300,336 (90.1%) did not have MI or CHD. The odds ratio of 0.432 indicates that participants who reported binge drinking were 56.8% less likely to have MI or CHD when compared to non-binge drinkers, as shown in Table 1.

**Table 1:** Prevalence of binge drinking in participants with self-reported MI or CHD *Significant. CI: confidence interval, OR: odds ratio, MI: myocardial infarction, CHD: coronary heart disease.

	MI or CHD			OR (CI)
Binge drinking	Yes	Yes	No	0.432* (0.4149,0.4507)
		2570 (4.5%)	53,969 (95.5%)	
	No	33,070 (9.9%)	300,336 (90.1%)	

The prevalence of MI or CHD among binge drinkers was 0.9% in those aged 18-24 years, 1.3% in those aged 25-44 years, 5.5% in those aged 45-64 years, and 14.6% in those aged 65 years and older. When gender was considered, MI or CHD was more prevalent in male binge drinkers, at 5.7%, compared with female binge drinkers, at 2.8%. About 4.6% of White binge drinkers had MI or CHD, 4.9% of Black binge drinkers had MI or CHD, and 3.5% of Hispanic binge drinkers had MI or CHD.

The likelihood of association between MI or CHD and binge drinking was 46.9% higher (OR=1.469) in those aged 18-24 years, 24.3% lower (OR=0.757) in those aged 25-44 years, 27.6% lower (OR=0.724) in those aged 45-64 years, and 18.8% lower (OR=0.812) in those aged 65 years and older. The likelihood of association between MI or CHD and binge drinking was 58.5% lower (OR=0.415) in males, and 65.3% lower (OR=0.347) in females. The likelihood of association between MI or CHD and binge drinking was 59.5% lower (OR=0.405) in White participants, 42.9% lower (OR=0.571) in Black participants, and 45.5% lower (OR=0.545) in Hispanic participants, as represented in Table 2.

**Table 2:** Prevalence of binge drinking in participants with self-reported MI or CHD based on demographic characteristics *Significant. CI: confidence interval, OR: odds ratio, MI: myocardial infarction, CHD: coronary heart disease.

Demographic characteristics	Binge drinking	N	Participants with MI or CHD	Participants without MI or CHD	OR (CI)
Age groups					
18-24 years	Yes	6122	57 (0.9%)	6065 (99.1%)	1.469* (1.0469, 2.041)
	No	17,615	112 (0.6%)	17,503 (99.4%)	
25-44 years	Yes	23,012	295 (1.3%)	22,717 (98.7%)	0.757 * (0.6633, 0.8611)
	No	70,535	1190 (1.7%)	69,345 (98.3%)	
45-64 years	Yes	19,668	1085 (5.5%)	18,583 (94.5%)	0.724* (0.678, 0.7733)
	No	110,203	8220 (7.5%)	101,983 (92.5%)	
65+ years	Yes	7737	1133 (14.6%)	6604 (85.4%)	0.812 * (0.7609,0.8668)
	No	135,053	23,548 (17.4%)	111,505 (82.6%)	
Gender					
Male	Yes	34,272	1942 (5.7%)	32,330 (94.3%)	0.415* (0.395,0.4352)
	No	148,808	18,827 (12.7%)	129,981 (87.3%)	
Female	Yes	22,267	628 (2.8%)	21,639 (97.2%)	0.347* (0.3195,0.3765)
	No	184,598	14,243 (7.7%)	170,355 (92.3%)	
Race					
White, non-Hispanic	Yes	41,547	1928 (4.6%)	39,619 (95.4%)	0.405* (0.3858, 0.4245)
	No	243,828	26,167 (10.7%)	217,661 (89.3%)	
Black, non-Hispanic	Yes	3253	158 (4.9%)	3095 (95.1%)	0.571* (0.4804, 0.6741)
	No	26,086	2142 (8.2%)	23,944 (91.8%)	
Hispanic/others	Yes	6331	222 (3.5%)	6109 (96.5%)	0.545* (0.4705, 0.628)
	No	30,344	1898 (6.3%)	28,446 (93.7%)	

Prevalence of MI or CHD in binge drinkers was 6.1% in participants with basic education and 3.9% in participants with advanced education. Prevalence of MI or CHD in binge drinkers was 8% in participants with an annual income of <$50,000, and 7.2% in participants with an annual income of >$50,000. The likelihood of association of MI or CHD in binge drinkers was 52.5% lower (OR=0.475) in participants with basic education and 58.9% lower (OR=0.411) in participants with advanced education. The likelihood of association of MI or CHD in binge drinkers was 48.5% lower (OR=0.515) in participants with income <$50,000 and 56.9% lower (OR=0.431) in participants with income >$50,000, as described in Table 3.

**Table 3:** Prevalence of binge drinking in participants with self-reported MI or CHD based on socioeconomic characteristics *Significant. CI: confidence interval, OR: odds ratio, MI: myocardial infarction, CHD: coronary heart disease.

Socioeconomic characteristics	Binge drinking	N	Participants with MI or CHD	Participants without MI or CHD	OR (CI)
Education level					
Basic education	Yes	16,023	983 (6.1%)	15,040 (93.9%)	0.475 * (0.4438, 0.5084)
	No	98,566	11,917 (12.1%)	86,649 (87.9%)	
Advanced education	Yes	40,373	1578 (3.9%)	38,795 (96.1%)	0.411* (0.39, 0.4333)
	No	233,627	21,032 (9%)	212,595 (91%)	
Annual income					
Less than $50,000	Yes	10,171	814 (8%)	9357 (92%)	0.515 * (0.4774, 0.5548)
	No	78,303	11,317 (14.5%)	66,986 (85.5%)	
Greater than $50,000	Yes	155,650	11,282 (7.2%)	144,368 (92.8%)	0.431* (0.4044, 0.459)
	No	10,171	814 (8%)	9357 (92%)	

## Discussion

The present study evaluates the association between binge drinking and the prevalence of MI or CHD using data from the BRFSS. A total of 389,945 participants were included in the analysis, with none excluded. Demographically, the study population was diverse, encompassing various age groups, genders, racial and ethnic backgrounds, educational levels, and income brackets. Statistical analysis revealed that binge drinkers had a significantly lower prevalence of self-reported MI or CHD compared to non-binge drinkers (OR: 0.432, 95% CI: 0.4149-0.4507). Protective associations persisted across demographic and socioeconomic subgroups, although the magnitude of effect varied. These findings suggest a complex interplay between binge drinking behaviors and cardiovascular health outcomes.

Comparison with prior studies reveals mixed findings. Some epidemiological studies align with our results, suggesting moderate alcohol consumption may exert a cardioprotective effect via mechanisms such as improved lipid profiles or reduced platelet aggregation [8]. However, other studies report a contrasting relationship, linking binge drinking with heightened cardiovascular risk due to increased oxidative stress, blood pressure variability, and endothelial dysfunction [9,10]. These discrepancies may stem from differences in population characteristics, definitions of binge drinking, and adjustments for confounders such as smoking and physical activity.

For instance, studies conducted in predominantly younger populations often highlight the adverse cardiovascular effects of binge drinking, possibly due to the acute hemodynamic stress associated with heavy episodic alcohol consumption [11]. In contrast, our findings in older participants suggest a protective trend, potentially reflecting survival bias or differing patterns of alcohol consumption among older adults. Another factor may be cultural variations in drinking behaviors; societies with predominantly moderate drinking habits may show more cardioprotective effects compared to those with patterns of excessive consumption [12]. For example, a review conducted by Ye et al. showed that cardiovascular risk factors in Chinese, Asian Indian, Filipino, and other Asian populations were associated with lower rates of binge drinking but relatively similar rates of cardiovascular disease, suggesting that alcohol may not be a strong associating factor for cardiovascular disease risk [12].

Gaps in the literature warrant further exploration. While this study suggests a significant association between binge drinking and reduced MI/CHD prevalence, the observational nature of our analysis limits causal inference. One area for exploration is the role of genetic polymorphisms in the greater context of alcohol consumption, as suggested by Hoek et al., whose review found that Mendelian randomized studies often yield inconclusive results [13]. Future research should examine the dose-response relationship between binge drinking frequency, quantity, and cardiovascular outcomes, ideally using longitudinal designs.

Limitations

This study has several limitations. The binary binge drinking definition, while standardized by the CDC, does not capture frequency or intensity. Reported odds ratios are crude, stratified estimates rather than adjusted models, and important confounders such as smoking, hypertension, and diabetes could not be included. This, along with reverse causation (sicker individuals abstaining), may explain the observed protective associations. As described by Wang et al., the cross-sectional design precludes any conclusions about causality [14]. Both binge drinking and cardiovascular outcomes were self-reported in BRFSS, raising the possibility of recall and misclassification bias. The study’s reliance on the BRFSS data limits generalizability, as the survey methodology excludes institutionalized individuals and those without landlines or cell phones, potentially underrepresenting marginalized populations [15]. Subgroup analyses were exploratory, lacked correction for multiple testing, and estimates in smaller groups may be unstable. Simplified education and income categories improved comparability but limited nuance. The survey also excluded institutionalized individuals and some low socioeconomic groups, and missing data were handled by case-wise exclusion, both of which may bias results. Finally, the cross-sectional design precludes causal inference. Despite these limitations, the use of a large, nationally representative dataset highlights important patterns and provides a basis for future longitudinal research.

## Conclusions

In this cross-sectional analysis of the 2022 BRFSS dataset, binge drinking was statistically associated with lower reported prevalence of MI and CHD across demographic and socioeconomic subgroups. These findings should not be interpreted as evidence of a true protective effect but rather as an observation that highlights the complexity of alcohol-cardiovascular interactions in population-level data. The results underscore the importance of considering residual confounding, reverse causation, and self-reporting biases when interpreting such associations. Despite these limitations, this study adds value by leveraging a large, nationally representative US dataset to identify unexpected patterns that warrant further investigation. Prospective studies are needed to clarify these associations and to better distinguish the impact of binge drinking from that of moderate alcohol consumption on cardiovascular outcomes.

## References

[1] Gerlich MG, Krämer A, Gmel G (2009). Patterns of alcohol consumption and acute myocardial infarction: a case-crossover analysis. Eur Addict Res.

[2] Tonelo D, Providência R, Gonçalves L (2013). Holiday heart syndrome revisited after 34 years. Arq Bras Cardiol.

[3] Ilic M, Grujicic Sipetic S, Ristic B, Ilic I (2018). Myocardial infarction and alcohol consumption: a case-control study. PLoS One.

[4] Leong DP, Smyth A, Teo KK (2014). Patterns of alcohol consumption and myocardial infarction risk: observations from 52 countries in the INTERHEART case-control study. Circulation.

[5] Patel A, Figueredo VM (2023). Alcohol and cardiovascular disease: helpful or hurtful. Rev Cardiovasc Med.

[6] (2024). CDC. About BRFSS. https://www.cdc.gov/brfss/about/index.htm.

[7] Bradway D (2019). Guidance on secondary analysis of existing data sets. https://ovpr.uconn.edu/services/rics/irb/researcher-guide/secondary-analysis-of-data-sets/.

[8] Mukamal KJ, Conigrave KM, Mittleman MA, Camargo CA Jr, Stampfer MJ, Willett WC, Rimm EB (2003). Roles of drinking pattern and type of alcohol consumed in coronary heart disease in men. N Engl J Med.

[9] Roerecke M, Rehm J (2012). The cardioprotective association of average alcohol consumption and ischaemic heart disease: a systematic review and meta-analysis. Addiction.

[10] Fernández-Solà J (2015). Cardiovascular risks and benefits of moderate and heavy alcohol consumption. Nat Rev Cardiol.

[11] O'Keefe JH, Bybee KA, Lavie CJ (2007). Alcohol and cardiovascular health: the razor-sharp double-edged sword. J Am Coll Cardiol.

[12] Ye J, Rust G, Baltrus P, Daniels E (2009). Cardiovascular risk factors among Asian Americans: results from a National Health Survey. Ann Epidemiol.

[13] Hoek AG, van Oort S, Mukamal KJ, Beulens JW (2022). Alcohol consumption and cardiovascular disease risk: placing new data in context. Curr Atheroscler Rep.

[14] Wang X, Cheng Z (2020). Cross-sectional studies: strengths, weaknesses, and recommendations. Chest.

[15] Lenzetta RL, Robbins E (2023). Behavioral risk factor surveillance system. StatPearls [Internet].

